# The impact of digital collaboration tools on inclusive leadership in multicultural teams in the context of global remote work: a psychological perspective on empathy, cohesion, and cross cultural communication

**DOI:** 10.3389/fpsyg.2026.1738857

**Published:** 2026-03-10

**Authors:** Xuan Yang, Youfu Wang

**Affiliations:** 1 School of Business Macau University of Science and Technology, Macau, China; 2 Faculty of Education, University of Cambridge, Cambridge, United Kingdom

**Keywords:** cross cultural communication, digital collaboration tools, empathy in leadership, inclusive leadership, multicultural teams, team cohesion

## Abstract

As remote and hybrid work becomes increasingly normalized, organizations rely on digital collaboration platforms to coordinate multicultural teams, yet it remains unclear how everyday technology-mediated interaction translates into inclusive leadership. This study examines whether digital collaboration tool use is associated with inclusive leadership through a sequential psychological pathway involving empathy, team cohesion, and cross-cultural communication. Drawing on social information processing theory and emotional contagion theory, survey data were collected from 240 employees working in multicultural remote or hybrid teams and analyzed using structural equation modeling and multi-group path analysis by gender. Results indicate that both the frequency of tool use and perceived interaction quality positively predict empathy in the full sample, and empathy is subsequently linked to stronger team cohesion, which supports more effective cross-cultural communication and, in turn, higher perceived inclusive leadership. Bootstrap analyses further support the significance of the sequential indirect effect. Multi-group comparisons reveal gender-related differences at the entry stage of the model: among women, tool use frequency predicts empathy whereas interaction quality is not significant; among men, both predictors are significant. Taken together, the findings suggest that the leadership relevance of digital collaboration tools operates primarily through socio-emotional and relational processes rather than through technology use alone, and that these processes may unfold differently across gender groups in multicultural remote work contexts.

## 1 Introduction

In the context of deepening globalization and accelerating digital transformation, remote work has gradually evolved from an emergency arrangement into a normalized mode of organizational operation across countries and regions. Since the COVID-19 pandemic, major economies such as the United States, the European Union, and Japan have successively institutionalized remote or hybrid work practices, and multinational enterprises have increasingly relied on digital platforms to organize geographically dispersed and culturally diverse virtual teams. In technology firms, consulting organizations, and platform-based enterprises, team members are often distributed across different countries and time zones, making digital collaboration tools the core infrastructure for everyday interaction. While this “boundaryless collaboration” model reduces spatial and temporal costs, it also amplifies managerial challenges rooted in cultural diversity and psychological distance, requiring leaders to address not only physical separation but also collaboration barriers arising from differences in cognitive styles, communication norms, and value orientations (Romano et al., 2007). Against this practical backdrop, scholarly research on multicultural teams and leadership has undergone a gradual evolution. Early studies primarily focused on structural and cultural differences in cross-cultural teams and generally treated technology as a neutral medium for information transmission and task coordination. With the widespread adoption of remote work and virtual collaboration, leadership research increasingly shifted toward the role of leadership behaviors in managing diversity, with inclusive leadership—characterized by respect for differences, openness to diversity, and the empowerment of individual members—being recognized as an important leadership approach for fostering psychological safety and collective cohesion (Shittu, 2022). Subsequent studies further emphasized that inclusive leadership is not merely a set of managerial practices but an affective and relational form of leadership, making it particularly suitable for remote and virtual team contexts where face-to-face interaction is limited. At the same time, the widespread use of digital collaboration tools such as Slack, Microsoft Teams, and Zoom has prompted scholars to recognize that these platforms are not only tools for task coordination but also important environments for emotional exchange, social sense-making, and cultural connection within organizations (Cagiltay et al., 2005). Despite these advances, significant gaps remain in the existing literature. Research on digital collaboration tools continues to be dominated by efficiency-oriented perspectives that emphasize information flow, coordination speed, and task performance, offering limited insight into how such tools shape leadership perceptions through psychological processes (Usama et al., 2025). Although recent studies have begun to acknowledge the social and relational functions of digital platforms, systematic empirical investigations into the psychological mechanisms linking digital collaboration to leadership outcomes remain scarce (Sivasankaran Balasubramaniam et al., 2024). Similarly, research on inclusive leadership has largely concentrated on institutional arrangements or observable leader behaviors, paying insufficient attention to how empathy, psychological safety, and relational alignment are cultivated in digitally mediated, multicultural environments (Amayo et al., 2023). Research on remote teams, while providing valuable insights into team adaptation processes, has likewise devoted relatively little attention to how leaders recalibrate emotional influence and relational norms under conditions of technology-mediated interaction (Isi et al., 2023). In response to these limitations, the present study conceptualizes digital collaboration tools as socio-psychological environments rather than neutral communication channels and examines how the use of such tools influences inclusive leadership through a sequential psychological mechanism involving empathy, team cohesion, and cross-cultural communication.

## 2 Literature review

### 2.1 Collaboration challenges and research progress of multicultural teams in the context of global remote work

With the rapid advancement of information and communication technologies and the deepening of globalization, remote work has gradually evolved from an emergency response into a routine organizational practice, particularly within multinational corporations and culturally diverse organizations (Romano et al., 2007). While virtual collaboration has effectively reduced geographical constraints and enhanced cross-regional information exchange, it has simultaneously introduced new layers of complexity into team coordination and relational dynamics. Prior research consistently indicates that cultural differences, communication barriers, and psychological distance remain among the most persistent challenges confronting multicultural remote teams (Samadi and Nixon, 2024). In technology-mediated environments, linguistic misunderstandings, divergent communication styles, and constraints on emotional expression are often amplified rather than alleviated, which in turn undermines trust formation and the stability of cooperative relationships (Yashan, 2024). Hurley and Shumway (2015) further argue that in the absence of face-to-face interaction, effective teamwork relies heavily on structured communication processes and clearly articulated shared goals; yet even under such conditions, relational cohesion may remain fragile. Emerging scholarship has therefore drawn attention to the vulnerability of team cohesion and psychological safety in remote and multicultural contexts. Rothouse (2020) notes that members from different cultural backgrounds frequently hold divergent interpretations of collaboration norms and role expectations, and without effective cultural mediation, these differences can weaken relational ties and reduce collective efficiency. Similarly, Calvert (2018) observes that when leaders fail to integrate diversity and inclusion principles into efforts to foster collaborative cultures, internal tensions may be intensified rather than mitigated. In response to these challenges, recent studies have increasingly emphasized the relevance of inclusive leadership in hybrid and remote teams. Hincapie and Costa (2024), for example, contend that leaders who cultivate empathy, cultural sensitivity, and psychological safety are better positioned to enhance collaboration quality and organizational performance in virtual settings. Despite these advances, existing research remains limited in explaining how cultural differences are enacted through digital platforms and how leaders may leverage everyday technology mediated interactions to shape the psychological mechanisms underpinning effective teamwork. This gap suggests the need for a more process-oriented examination of how digital collaboration tools interact with individual-level psychological resources and relational dynamics to support inclusive leadership and sustained team functioning in multicultural remote environments.

### 2.2 Theoretical evolution of inclusive leadership and its adaptation challenges in the digital era

Inclusive leadership was originally conceptualized in organizational contexts characterized by face to face interaction and physically co located management structures, with a central emphasis on open communication, cultural sensitivity, and empowering behaviors as foundations for trust and psychological safety among team members (Williams, 2008). As organizations undergo deep digital transformation and remote and hybrid work arrangements become increasingly normalized, this leadership model faces substantial challenges in adapting to technology mediated environments. In virtual settings, leader member interaction is no longer anchored in embodied cues such as body language or immediate affective feedback but is instead filtered through text based, audio, and video channels. This decontextualized mode of communication reduces opportunities for spontaneous emotional recognition and empathic exchange, thereby complicating the transmission and interpretation of inclusive signals (Cagiltay et al., 2005). Research on multicultural virtual teams suggests that digital platforms may even unintentionally reinforce existing power asymmetries, limiting the willingness or ability of culturally marginalized members to voice authentic perspectives and constraining the development of genuinely inclusive interaction norms. In a similar vein, Caputo et al. (2024) argue that leadership approaches centered primarily on task coordination and control are increasingly insufficient in hybrid work contexts, where cultural diversity and virtual collaboration intensify relational ambiguity. Against this backdrop, inclusive leadership, with its focus on relationship building and emotional support, has been positioned as a critical driver of cohesion and adaptability in multicultural teams. However, this shift involves more than a stylistic adjustment; it requires leaders to reconstruct trust, empathy, and psychological safety under conditions of technological mediation. Asfahani (2025) notes that in remote work environments, leaders equipped with cultural intelligence and cross cultural communication competence are better able to stimulate innovation and sustain an inclusive climate, indicating that inclusiveness should be understood not as a stable personality attribute but as a context dependent capability shaped by interactional conditions and supported by digital tools. At a broader level, Cortellazzo et al. (2019) observe that digitalization is transforming leadership functions toward psychological visibility, value resonance, and boundary spanning, introducing simultaneous technological and emotional challenges to inclusive practice. Communication delays, fragmented feedback, and asymmetric participation can weaken traditional listening based strategies, making the cultivation of psychological safety more fragile in virtual teams. Within this evolving organizational landscape, Jensen (2022) further demonstrates that leaders seeking to foster innovation and cultural inclusiveness in digitally connected environments must integrate technological competence with cultural sensitivity and situational judgment. These developments indicate that inclusive leadership is undergoing a transition from an interpersonally grounded model toward one that is technologically adaptive, where leaders’ ability to recognize cultural differences accurately and maintain emotional connection through digital interaction becomes central. Such conditions underscore the need to reconceptualize the psychological mechanisms underlying inclusive leadership and to examine how they operate at the intersection of remote collaboration, digital mediation, and multicultural interaction, thereby providing a more precise foundation for the sequential mechanisms proposed in the present study.

### 2.3 Organizational functions and research biases of digital collaboration tools: from efficiency orientation to psychological mechanisms

As remote work has become an established organizational norm, digital collaboration tools such as Zoom, Slack, and Microsoft Teams have been widely adopted as core infrastructures for communication and project coordination across multinational organizations. However, a substantial portion of existing research continues to approach these tools primarily from an efficiency-oriented perspective, emphasizing task coordination, information processing, and workflow optimization while paying comparatively limited attention to their psychological and relational implications (Mäkilouko, 2004). This efficiency-dominant orientation implicitly treats digital platforms as neutral technological instruments rather than as socially consequential environments (Steers et al., 2013). Empirical studies of cross-functional and remote project teams further indicate that collaboration difficulties often persist even when technological efficiency is high, suggesting limits to purely process-oriented explanations (Sivasankaran Balasubramaniam et al., 2024). More recent research has begun to challenge this instrumental view by showing that digital collaboration tools also function as social and affective spaces. For instance, digitally mediated interaction has been shown to transmit informal feedback and leaders’ emotional cues, thereby contributing to team members’ sense of belonging and psychological safety in culturally diverse teams (Erfan, 2024). From a leadership perspective, inclusive leadership theory emphasizes that relational sensitivity and openness to difference are central to fostering trust in multicultural contexts (Williams, 2008). Building on this view, recent work highlights that inclusive leadership becomes particularly salient in culturally diverse and digitally mediated environments where relational signals are easily attenuated (Anna Karadencheva, 2025).

At the micro-interaction level, empathic communicative behaviors embedded in digital platforms, such as acknowledgment and supportive responses, have been found to enhance perceptions of inclusive leadership (Nakamura and Milner, 2023). Related evidence from educational and training contexts similarly suggests that digital platforms can serve as environments for cultivating cultural awareness rather than merely channels for task delivery (Imbrie et al., 2021). Studies on multicultural team interaction further indicate that cultural differences continue to shape communication patterns and cohesion in digital settings, even when technological access is equal (Samadi and Nixon, 2024). Despite these insights, much of the cross-cultural management literature still treats technology as a contextual backdrop rather than as an active force shaping trust formation and relational dynamics (Iriogbe et al., 2024). Research on international and remote leadership similarly notes that leadership effectiveness in virtual multicultural teams cannot be fully explained by formal structures or technological arrangements alone (Makhmudova, 2024). In response to these limitations, scholars have called for a reconceptualization of digital collaboration technologies as embedded within broader socio-psychological systems that influence leadership processes (Cortellazzo et al., 2019). Recent empirical work further supports this shift by demonstrating that empathetic leadership plays a particularly important role in shaping employee experiences in remote work environments (Kurniawan et al., 2025).

### 2.4 Fragmented research on empathy, cohesion, and cross cultural communication in virtual contexts

As global remote collaboration becomes increasingly normalized, empathy, team cohesion, and cross-cultural communication are widely recognized as critical psychological conditions for sustaining diverse virtual teams, yet the existing literature remains fragmented and weakly integrated, with most studies examining these mechanisms in isolation and thereby limiting a coherent understanding of how inclusive leadership emerges through their interaction in multicultural digital contexts. Early work on empathy, most notably Broome’s relational empathy framework, emphasized that in intercultural interaction, recognizing and engaging with difference is more consequential than seeking similarity, offering a foundational critique of cognition-centered models that assume emotional alignment through homogeneity (Broome, 1991). Building on this perspective, subsequent research has extended relational empathy into digitally mediated environments, demonstrating that empathic understanding can still be cultivated despite the absence of face-to-face interaction; for example, Zhou et al. (2025) showed, through virtual ethnographic evidence, that relational empathy emerges through repeated interaction, emotional exchange, and reflective dialogue in cross-cultural online settings. Nevertheless, despite these conceptual advances, systematic empirical examination of empathy as an active psychological mechanism in virtual teamwork remains limited, particularly with respect to how empathic understanding translates into collective relational outcomes. A similar pattern of fragmentation characterizes research on team cohesion, where studies on multicultural teams often prioritize macro-level processes such as cultural adaptation or value alignment while devoting relatively little attention to how cohesion is constructed through everyday interaction and emotional experience within digital collaboration platforms (Steers et al., 2013). Although psychological safety and cohesion are widely acknowledged as essential for effective collaboration, they are frequently treated as outcomes of structural arrangements or leadership prescriptions rather than as emergent properties of ongoing interaction shaped by fragile trust and identity ambiguity in multicultural contexts (Rothouse, 2020). Research on cross-cultural communication further illustrates this gap between relational processes and technological mediation, as much of the literature remains focused on system design, media richness, or interaction efficiency rather than on how relational meaning, trust, and emotional safety are constructed through communication itself (Kotis et al., 2020). Studies emphasizing ICT proficiency highlight its role in enabling cross-boundary interaction, yet offer limited insight into how cultural differences are translated into communicative styles and how trust is socially negotiated through digitally mediated exchange (Sayogo et al., 2011). More recent scholarship has begun to challenge this separation between technology and psychology by questioning decontextualized approaches to cultural intelligence; for instance, Pacheco (2024) argues that communication challenges in remote collaboration cannot be adequately addressed through standardized training models alone but must be understood within specific organizational and technological contexts. From a complementary human resource development perspective, research has emphasized cross-cultural competence and diversity-oriented training as foundations for inclusion, while leaving unresolved the question of how such competencies are enacted and sustained through routine digital interaction (Mouboua et al., 2024). Collectively, this body of research points to a critical theoretical gap: the absence of an integrated perspective that conceptualizes empathy, team cohesion, and cross-cultural communication as sequential and mutually reinforcing mechanisms rather than as independent attributes. Addressing this gap directly motivates the present study, which conceptualizes these constructs as a linked psychological pathway through which digital collaboration shapes inclusive leadership in multicultural remote teams.

### 2.5 Research gaps and theoretical integration: building a multi level mechanism linking technology, psychology, and leadership

Although inclusive leadership has become an increasingly prominent concept in research on remote and multicultural organizations, the existing literature remains fragmented in both theoretical focus and analytical level. Prior studies have demonstrated that inclusive cultures enhance organizational performance and that inclusive leadership plays a critical role in leveraging workforce diversity, particularly in culturally heterogeneous settings (Williams, 2008; Tiwari, 2025). However, much of this work is anchored at the macro level of values, norms, or institutional arrangements, offering limited insight into how inclusive leadership is enacted and sustained through everyday interaction in remote teams, where leadership signals are largely mediated by digital technologies rather than face-to-face contact (Cortellazzo et al., 2019). In parallel, research on digital collaboration technologies has emphasized their role in enabling coordination, information sharing, and perceived connectedness among geographically dispersed members (Romano et al., 2007; Hincapie and Costa, 2024), yet technological factors are still predominantly treated as external enablers of collaboration rather than as integral components embedded within leadership and relational processes (Steers et al., 2013). Emerging psychological perspectives have begun to link empathic interaction with trust formation and leadership perception in virtual contexts (Nakamura and Milner, 2023), but these frameworks often overlook the cultural heterogeneity and platform-specific conditions that characterize multicultural remote collaboration, thereby limiting their explanatory scope, particularly with respect to how psychological mechanisms unfold through digitally mediated interaction (Cagiltay et al., 2005). As a result, the current literature tends to present partial and disconnected linkages between technology and coordination, psychology and leadership, or culture and communication, without offering a coherent multilevel explanation that integrates digital collaboration, psychological mechanisms, and inclusive leadership outcomes within a single analytical framework. Addressing this gap, the present study conceptualizes digital collaboration tools not as neutral infrastructures, but as psychologically consequential interaction environments through which empathy, team cohesion, and cross-cultural communication are sequentially activated and translated into perceptions of inclusive leadership in multicultural remote teams, and on this basis, the following hypotheses are proposed:

*H1*: frequent use of digital collaboration tools strengthens leaders’ empathy;

*H2*: empathy enhances team cohesion;

*H3*: team cohesion facilitates effective cross-cultural communication;

*H4*: cross-cultural communication contributes to perceptions of inclusive leadership;

*H5*: empathy mediates the relationship between tool use and inclusive leadership; and H6 team cohesion and cross-cultural communication jointly form a sequential mediating pathway in this mechanism.

## 3 Methods

### 3.1 Measurement development and scale justification

The measures for digital collaboration tool use frequency and interaction quality were self-developed to capture key features of technology-mediated collaboration that are not fully addressed by existing standardized scales. Item development was theoretically grounded in prior research on digital communication intensity and the use of remote work technologies, with item content aligned to commonly examined indicators of platform engagement and interaction experiences in virtual collaboration settings (Romano et al., 2007). Tool use frequency reflects the regularity and breadth of individuals’ engagement with commonly used digital collaboration tools in their daily work activities. Interaction quality captures respondents’ perceptions of emotional responsiveness, clarity of communication, and relational tone during digitally mediated interactions, reflecting the socio-psychological nature of collaboration in virtual environments (Usama et al., 2025).

To ensure content validity and conceptual clarity, all measurement items were systematically reviewed and refined prior to formal data collection through an expert evaluation process. Two researchers with expertise in organizational psychology and leadership studies participated in this process, assessing the extent to which each item accurately represented its intended theoretical construct and evaluating the clarity, consistency, and appropriateness of item wording in multicultural and geographically distributed work contexts. Particular attention was paid to minimizing culturally specific expressions and potential semantic ambiguities to ensure that respondents from diverse cultural backgrounds could interpret the items in a consistent manner. Based on the experts’ feedback, minor revisions were made to improve wording precision prior to the administration of the formal survey. In terms of measurement format, the study adopted a hybrid scale design that combines a Likert-type measurement framework with a slider-based response interface. Specifically, items were presented using a slider format in which only the midpoint of the scale was explicitly labeled, while the remaining positions were left unlabeled. This design allowed respondents to freely adjust the slider to a position that best reflected their genuine subjective perceptions. For analytical purposes, slider positions were linearly mapped onto a 7-point Likert scale ranging from 1 to 7. This approach preserves the theoretical structure and comparability of traditional Likert scales while reducing anchoring effects associated with fixed numerical labels, thereby encouraging more nuanced and intuitive responses and enhancing the sensitivity and ecological validity of the measurements.

### 3.2 Data collection procedure and paired design

The total sample consisted of 240 participants, all of whom were members of multicultural remote or hybrid collaboration teams. Participants were eligible for inclusion if they had at least 6 months of experience in remote collaboration, worked in teams composed of members from at least two distinct cultural backgrounds, and regularly used at least one digital collaboration platform, such as Slack, Zoom, or Microsoft Teams. Participants were recruited through online professional platforms, including LinkedIn and remote work communities, which enabled access to geographically dispersed employees across multiple organizations and industries. Of the valid responses collected, the final dataset comprised 121 women and 119 men. All participants were engaged in remote or hybrid collaboration as part of their regular work arrangements, allowing the study to capture substantial variation in collaboration practices and leadership perceptions across organizational contexts. Data were collected using a standardized online questionnaire, with an average completion time of approximately 10–15 min. Participation was voluntary, and all respondents provided electronic informed consent prior to participation. Respondents were informed that their responses would remain anonymous and would be used solely for academic research purposes.

The survey design was framed around leader–member interaction contexts, whereby respondents were instructed to answer all questionnaire items based on their direct interaction experiences with their immediate leaders within the same team. To reduce social desirability effects and response dependency, participants completed the questionnaires independently without time pressure. Although the questionnaire referenced leader–member interaction contexts, all data were collected at the individual team member level rather than as matched dyads. Each response therefore represents an individual member’s perception of inclusive leadership and related psychological mechanisms embedded within a shared team context. Accordingly, the reported sample size and gender distribution refer to individual respondents rather than paired leader–member observations, and all analyses were conducted at the individual level. Team identifiers were included to ensure that respondents consistently referred to the same team context when completing the questionnaire. In addition, because responses were collected using a slider-based format, the recorded scores were treated as continuous values; each slider position was linearly rescaled to a 1–7 metric and retained as a continuous score (with decimal precision preserved) for all subsequent analyses.

### 3.3 Control of common method bias

Several procedural and design-related remedies were implemented to reduce the risk of common method bias. First, respondent anonymity was guaranteed, and participants were explicitly informed that there were no correct or incorrect answers, thereby reducing social desirability concerns. Second, questionnaire items were randomly ordered to minimize systematic response tendencies. Third, the questionnaire design conceptually separated technology-related predictors, psychological mediators, and leadership outcome variables, which reduces respondents’ tendency to infer artificial causal relationships. In addition to these procedural controls, Harman’s single-factor test was conducted. The results showed that no single factor accounted for the majority of total variance, indicating that common method bias was unlikely to pose a serious threat to the validity of the results.

### 3.4 Analytical strategy and model selection rationale

Structural equation modeling (SEM) was employed to test the proposed theoretical model because it allows for the simultaneous estimation of multiple relationships among latent constructs while accounting for measurement error. This approach is particularly suitable for examining complex psychological mechanisms involving multiple mediating processes. Sequential mediation analysis was conducted to test the hypothesized psychological pathway through which digital collaboration tool use influences inclusive leadership via empathy, team cohesion, and cross-cultural communication. Multi-group analysis was further performed to examine potential gender differences in the structural relationships, as prior research indicates that leadership perception and emotional processing may vary across gender groups in digital work contexts.

### 3.5 Indicator system

The measurement framework was structured across four dimensions—technological, psychological, relational, and leadership—as summarized in Table 1. The technological dimension distinguishes between frequency of collaboration tool use and perceived interaction quality, capturing both behavioral intensity and experiential evaluation. Psychological and relational mechanisms are represented by relational empathy, team cohesion, and cross-cultural communication effectiveness, which function as key mediating pathways. Perceived inclusive leadership serves as the core outcome variable. All constructs were conceptually aligned with established theoretical foundations to ensure coherence between the analytical framework and measurement design.

**Table 1:** Multidimensional indicator system and measurement design.

Primary dimension	Secondary indicator	Theoretical foundation	Data source (with example items)
Technological dimension	Frequency of collaboration tool use	The intensity of tool use serves as a prerequisite for activating psychological pathways and leadership interaction mechanisms.	Self-developed scale using a 7-point slider-based Likert format (e.g., “I frequently use digital collaboration tools such as Zoom, Slack, or Teams in my daily work”).
Technological dimension	Quality of collaborative interaction	Emotional responsiveness within digital communication shapes inclusive cognition; digital platforms facilitate multicultural communication.	Questionnaire survey using a 7-point slider-based Likert format (e.g., “Interactions through remote collaboration platforms feel emotionally responsive and supportive”).
Psychological dimension	Leaders’ empathy level	The relational empathy model applies to cross-cultural remote collaboration contexts.	Relational Empathy Scale (e.g., “My leader understands how cultural differences influence team members’ feelings in remote work settings”).
Psychological dimension	Team psychological safety	Trust and psychological safety among team members constitute the foundation of team cohesion.	Team Cohesion Scale – psychological safety dimension (e.g., “Team members feel safe expressing their opinions during remote collaboration”).
Relational dimension	Team cohesion	In multicultural settings, team cohesion is jointly influenced by communication quality and identity alignment.	Team Cohesion Scale (e.g., “Members of my team trust one another when working together remotely”).
Relational dimension	Effectiveness of cross-cultural communication	Digital media serve as cultural bridges that enhance cross-cultural understanding and collaboration.	Cross-Cultural Communication Scale (e.g., “Communication with team members from different cultural backgrounds is clear and effective”).
Leadership dimension	Perceived inclusive leadership	Inclusive leadership centers on empowerment, open communication, and cultural sensitivity.	Inclusive Leadership Questionnaire (e.g., “My leader encourages participation from team members with diverse cultural backgrounds”).
Leadership dimension	Leaders’ cultural intelligence and digital literacy	Leadership effectiveness in digital contexts depends on both cultural intelligence and media literacy.	Self-assessment items and optional open-ended questions (e.g., “I am confident in leading culturally diverse teams through digital collaboration tools”).
Technological dimension	Frequency of collaboration tool use	The intensity of tool use serves as a prerequisite for activating psychological pathways and leadership interaction mechanisms.	Self-developed scale using a 7-point slider-based Likert format (e.g., “I frequently use digital collaboration tools such as Zoom, Slack, or Teams in my daily work”).

### 3.6 Variable description

The operational definitions and measurement specifications of all variables are presented in Table 2. Independent variables reflect technological engagement and interaction experience in remote collaboration contexts, mediating variables capture the underlying psychological and relational mechanisms, and perceived inclusive leadership constitutes the dependent variable. Gender, years of remote work experience, and team cultural diversity ratio were included as control variables to account for structural and demographic variation. All continuous constructs were measured using 7-point Likert-type scales.

**Table 2:** Variable definitions, conceptual meanings, and measurement scales.

Variable type	Variable name	Definition and conceptual meaning	Measurement and scale
Independent variables	Frequency of collaboration tool use	Refers to the frequency and breadth with which remote workers use platforms such as Zoom, Slack, and Teams, reflecting their level of technological engagement.	Self developed scale (1–7 points, frequency items)
	Quality of collaborative interaction	Captures participants’ overall perception of the emotional responsiveness, interaction quality, and non task communication that occur through remote collaboration platforms.	Self developed Likert scale (1–7 points)
Mediating variables	Relational empathy	Refers to a leader’s ability to perceive others’ situations, cultural differences, and emotional states in a remote multicultural environment; it represents the core psychological mechanism underlying inclusive leadership.	Relational Empathy Scale (Zhou et al., 2025)
	Team cohesion	Denotes the level of trust, willingness to collaborate, and emotional connection among team members an essential factor facilitating cross cultural understanding.	Team Cohesion Scale (Rothouse, 2020)
	Effectiveness of cross cultural communication	Refers to the accuracy, mutual understanding, and fluency of information exchange among team members from different cultural backgrounds.	Cross Cultural Communication Scale
Dependent variable	Perceived inclusive leadership	Represents team members’ subjective evaluation of whether their leader demonstrates respect for diversity, fosters inclusion, and encourages participation; it is the core outcome variable of this study.	Inclusive Leadership Questionnaire
Control variables	Gender	Participant’s gender, categorized as “male” or “female.”	Self reported (categorical variable)
	Years of remote work experience (remote experience years)	The number of years participants have engaged in remote collaboration, which may influence their perceptions of technology use and leadership.	Self reported (continuous variable)
	Team cultural diversity ratio (culture diversity ratio)	The proportion of team members from different countries or regions, reflecting the level of cultural diversity that may shape cross cultural understanding and inclusiveness.	Self assessed percentage (0–100%)

## 4 Results

### 4.1 Descriptive statistics

As shown in Table 3, respondents reported an average of 3.68 years of remote work experience (SD = 2.68), reflecting notable variation in prior exposure to remote collaboration. Tool usage frequency exhibited a relatively high mean value (*M* = 5.69 on a 7-point scale), with the interquartile range concentrated between 5.0 and 6.0, indicating that frequent use of digital collaboration platforms was common among participants. By comparison, the mean score for interaction quality was slightly lower (*M* = 5.30), suggesting that frequent tool use did not uniformly correspond to higher evaluations of interaction effectiveness. With respect to psychological and relational variables, empathy (*M* = 5.65), team cohesion (*M* = 5.40), cross-cultural communication (*M* = 5.37), and inclusive leadership (*M* = 5.47) all demonstrated relatively high average levels, accompanied by moderate dispersion, with standard deviations ranging from 0.78 to 0.88. These distributions reflect generally positive perceptions across the key constructs examined in multicultural remote teams, while also indicating meaningful variability across respondents. Notably, the minimum observed value for inclusive leadership was 2.60, pointing to substantial differences in perceived inclusiveness across team contexts. Taken together, the descriptive patterns indicate that although the use of remote collaboration technologies was prevalent, variation persisted in evaluations of interaction quality and leadership-related experiences, and the relatively limited dispersion across most variables suggests a degree of homogeneity within the sample, highlighting the relevance of subsequent analyses for examining relational structures and subgroup-level differences.

**Table 3:** Descriptive statistics of key variables (*n* = 240).

Variable	Mean	Std. Dev.	Min	25th Percentile	50th Percentile	75th Percentile	Max
Remote experience (yrs)	3.68	2.68	0	1	3	5	13
Tool use frequency	5.69	0.87	3	5	6	6	7
Interaction quality	5.3	0.9	2.75	4.75	5.5	6	7
Empathy	5.65	0.78	3.8	5.2	5.8	6.2	7
Team cohesion	5.4	0.81	3	4.8	5.4	6	7
Cross cultural comm.	5.37	0.88	3	4.8	5.4	6	7
Inclusive leadership	5.47	0.85	2.6	5	5.6	6	7

### 4.2 Reliability and validity analysis

Table 4 reports the reliability and validity statistics for the measurement scales used in this study. All constructs demonstrated satisfactory internal consistency, with Cronbach’s *α* values ranging from 0.824 to 0.921, exceeding the commonly accepted threshold of 0.70. The overall scale also exhibited excellent reliability, with a Cronbach’s α of 0.949. The Kaiser–Meyer–Olkin (KMO) measure of sampling adequacy was 0.932, indicating that the data were highly suitable for factor-based analysis. Bartlett’s test of sphericity was statistically significant (*χ*^2^ = 3684.31, df = 153, *p* < 0.001), suggesting that the correlation matrix significantly differed from an identity matrix and that sufficient inter-item correlations existed. Collectively, these results confirm that the measurement model met established criteria for reliability and construct validity and was appropriate for the subsequent analyses.

**Table 4:** Reliability and validity tests.

Measure	Items	Value	Conclusion
Tool use frequency	TUF1, TUF2, TUF3	Cronbach’s *α* = 0.883	Reliable
Interaction quality	IQ1, IQ2, IQ3	Cronbach’s *α* = 0.921	Reliable
Empathy	EMP1, EMP2, EMP3	Cronbach’s *α* = 0.908	Reliable
Team cohesion	TC1, TC2, TC3	Cronbach’s *α* = 0.857	Reliable
Cross-cultural communication	CCC1, CCC2, CCC3	Cronbach’s *α* = 0.824	Reliable
Inclusive leadership	IL1, IL2, IL3	Cronbach’s *α* = 0.860	Reliable
Overall scale	18 items	Cronbach’s *α* = 0.949	Reliable
KMO	–	0.932	Adequate
Bartlett’s test of sphericity	–	*χ*^2^ = 3684.31 (df = 153), *p* < 0.001	Significant

### 4.3 Pearson correlation analysis

Results from Table 5 indicate that all key variables were positively and significantly correlated. Tool usage frequency was strongly correlated with interaction quality (*r* = 0.853, *p* < 0.001) and empathy (*r* = 0.820, *p* < 0.001), and interaction quality was also positively associated with empathy (*r* = 0.781, *p* < 0.001). Empathy showed significant positive correlations with team cohesion (*r* = 0.590, *p* < 0.001) and inclusive leadership (*r* = 0.683, *p* < 0.001). Team cohesion was strongly related to both cross-cultural communication (*r* = 0.612, *p* < 0.001) and inclusive leadership (*r* = 0.707, *p* < 0.001). Cross-cultural communication was positively correlated with inclusive leadership (*r* = 0.525, *p* < 0.001). The magnitude of several correlations was relatively high, suggesting substantial shared variance among the constructs. These associations provide preliminary empirical support for the proposed relationships and indicate that the variables are systematically related, warranting further examination through mediation and structural modeling analyses.

**Table 5:** Pearson correlation matrix.

Variable	1	2	3	4	5	6
1. Tool usage frequency	1	0.853***	0.820***	0.538***	0.373***	0.600***
2. Interaction quality	0.853***	1	0.781***	0.449***	0.342***	0.549***
3. Empathy	0.820***	0.781***	1	0.590***	0.393***	0.683***
4. Team cohesion	0.538***	0.449***	0.590***	1	0.612***	0.707***
5. Cross cultural communication	0.373***	0.342***	0.393***	0.612***	1	0.525***
6. Inclusive leadership	0.600***	0.549***	0.683***	0.707***	0.525***	1

***p < 0.001.

### 4.4 Sequential mediation analysis

**Figure 1:**
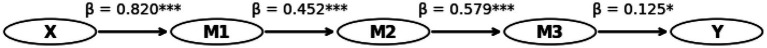
Chain mediation path diagram. *p < 0.05, **p < 0.01, ***p < 0.001.

As shown in Table 6 and Figure 1, the frequency of digital collaboration tool use was positively associated with empathy (*β* = 0.820, *p* < 0.001). Empathy was positively related to team cohesion (*β* = 0.452, *p* < 0.001), and team cohesion was positively associated with cross-cultural communication (*β* = 0.579, *p* < 0.001). Cross-cultural communication was also positively associated with inclusive leadership, with a smaller standardized coefficient (*β* = 0.125, *p* = 0.015). All estimated path coefficients were statistically significant. These results indicate that the associations among the study variables follow the sequential structure specified in the proposed model.

**Table 6:** Chain mediation analysis results.

Path	*β*	*p* value
X (Tool Usage Frequency) → M1 (Empathy)	0.820	<0.001***
M1 (Empathy) → M2 (Team Cohesion)	0.452	<0.001***
M2 (Team Cohesion) → M3 (Cross Cultural Communication)	0.579	<0.001***
M3 (Cross Cultural Communication) → Y (Inclusive Leadership)	0.125	0.015*

*p < 0.05, ***p < 0.001.

### 4.5 Robustness test

**Figure 2:**
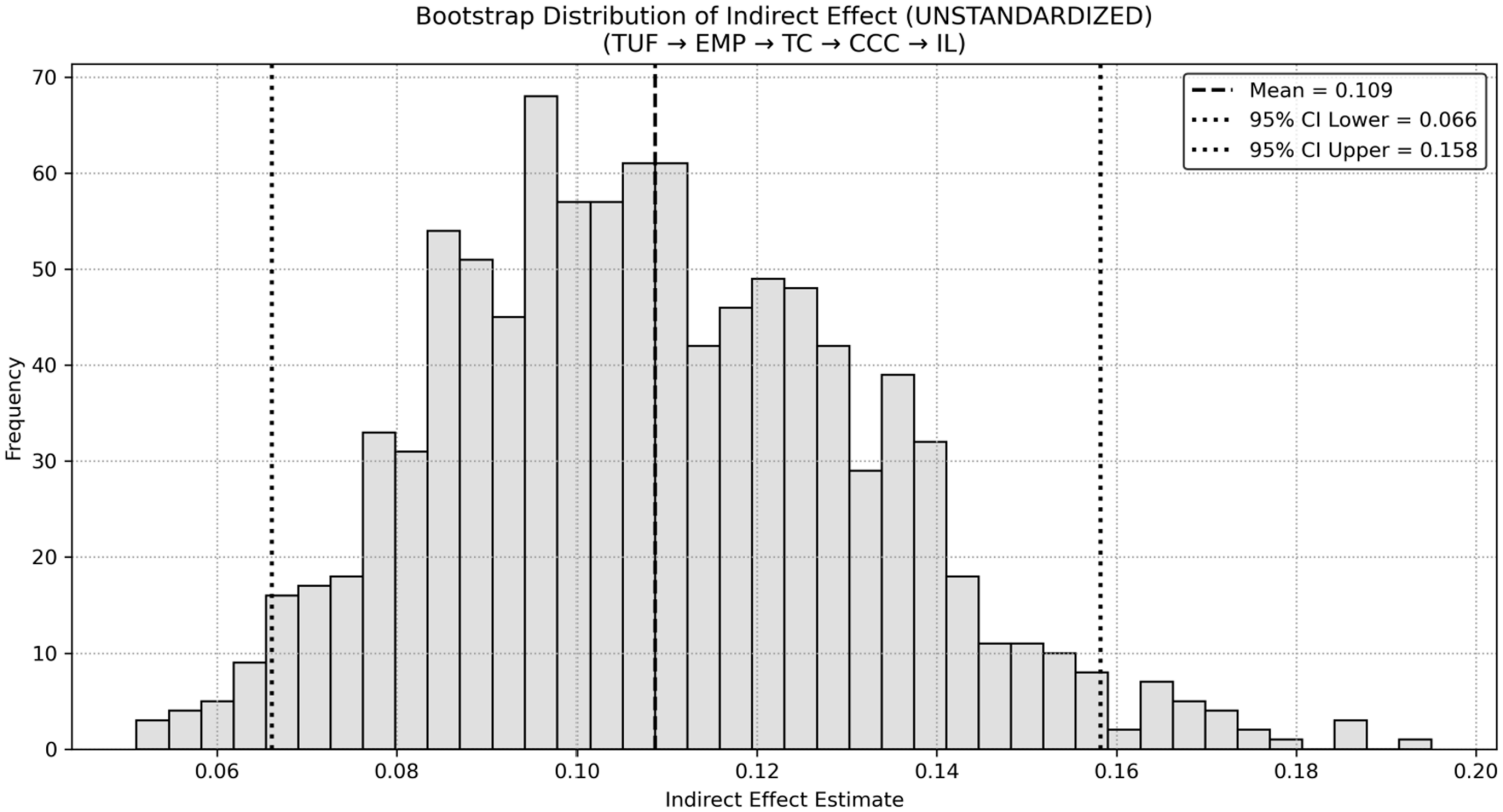
Bootstrap distribution of indirect effect estimates (1,000 resamples).

To assess the robustness of the sequential mediation pathway, a bootstrap resampling procedure with 1,000 samples was conducted. As reported in Table 7 and illustrated in Figure 2, the indirect effect of the pathway from tool usage frequency to inclusive leadership via empathy, team cohesion, and cross-cultural communication was 0.109, with a 95% confidence interval of [0.066, 0.158], which did not include zero (*p* < 0.001). These results indicate that the proposed sequential indirect effect is statistically robust and remains stable under resampling estimation.

**Table 7:** Bootstrap estimates of indirect effects (1,000 Samples).

Indirect pathway	Mean effect	95% CI lower	95% CI upper	Significance
Tool usage frequency → empathy → team cohesion → cross cultural communication → inclusive leadership	0.109	0.066	0.158	***

***p < 0.001.

### 4.6 SEM

**Figure 3:**
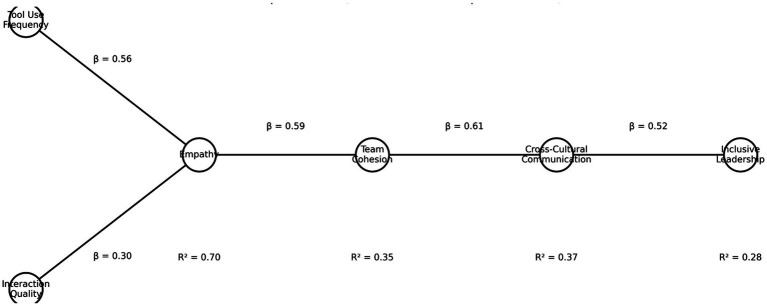
Structural equation model of tool use, empathy, communication, and inclusive leadership.

As reported in Table 8 and illustrated in Figure 3, both tool usage frequency (*β* = 0.563, *p* < 0.001) and interaction quality (*β* = 0.300, *p* < 0.001) were positively associated with empathy, jointly accounting for 69.6% of the variance in empathy. Empathy was positively associated with team cohesion (*β* = 0.590, *p* < 0.001), and team cohesion was positively related to cross-cultural communication (*β* = 0.612, *p* < 0.001). Cross-cultural communication, in turn, showed a positive association with inclusive leadership (*β* = 0.525, *p* < 0.001). The explained variance was 34.8% for team cohesion, 37.5% for cross-cultural communication, and 27.5% for inclusive leadership. Model fit indices indicated an acceptable fit to the data (*χ*^2^ = 22.351, df = 12, *χ*^2^/df = 1.863, CFI = 0.982, TLI = 0.974, RMSEA = 0.060, SRMR = 0.041). These results show that the estimated structural relationships among the variables were statistically supported within the specified model.

**Table 8:** Structural equation model (SEM) results.

Path/Index	Std. *β*	*t* value	*p* value	*R*^2^/value	Significance
Tool usage frequency → empathy	0.563	8.203	< 0.001	*R*^2^ (empathy) = 0.696	***
Interaction quality → empathy	0.300	4.376	< 0.001	*R*^2^ (empathy) = 0.696	***
Empathy → team cohesion	0.590	11.260	< 0.001	*R*^2^ (team cohesion) = 0.348	***
Team cohesion → cross-cultural communication	0.612	11.945	< 0.001	*R*^2^ (cross-cultural communication) = 0.375	***
Cross-cultural communication → inclusive leadership	0.525	9.509	< 0.001	*R*^2^ (inclusive leadership) = 0.275	***
Model fit indices				*χ*^2^ = 22.351, df = 12, *χ*^2^/df = 1.863, CFI = 0.982, TLI = 0.974, RMSEA = 0.060, SRMR = 0.041, N = 240	

***p < 0.001.

### 4.7 Multi group analysis

**Figure 4:**
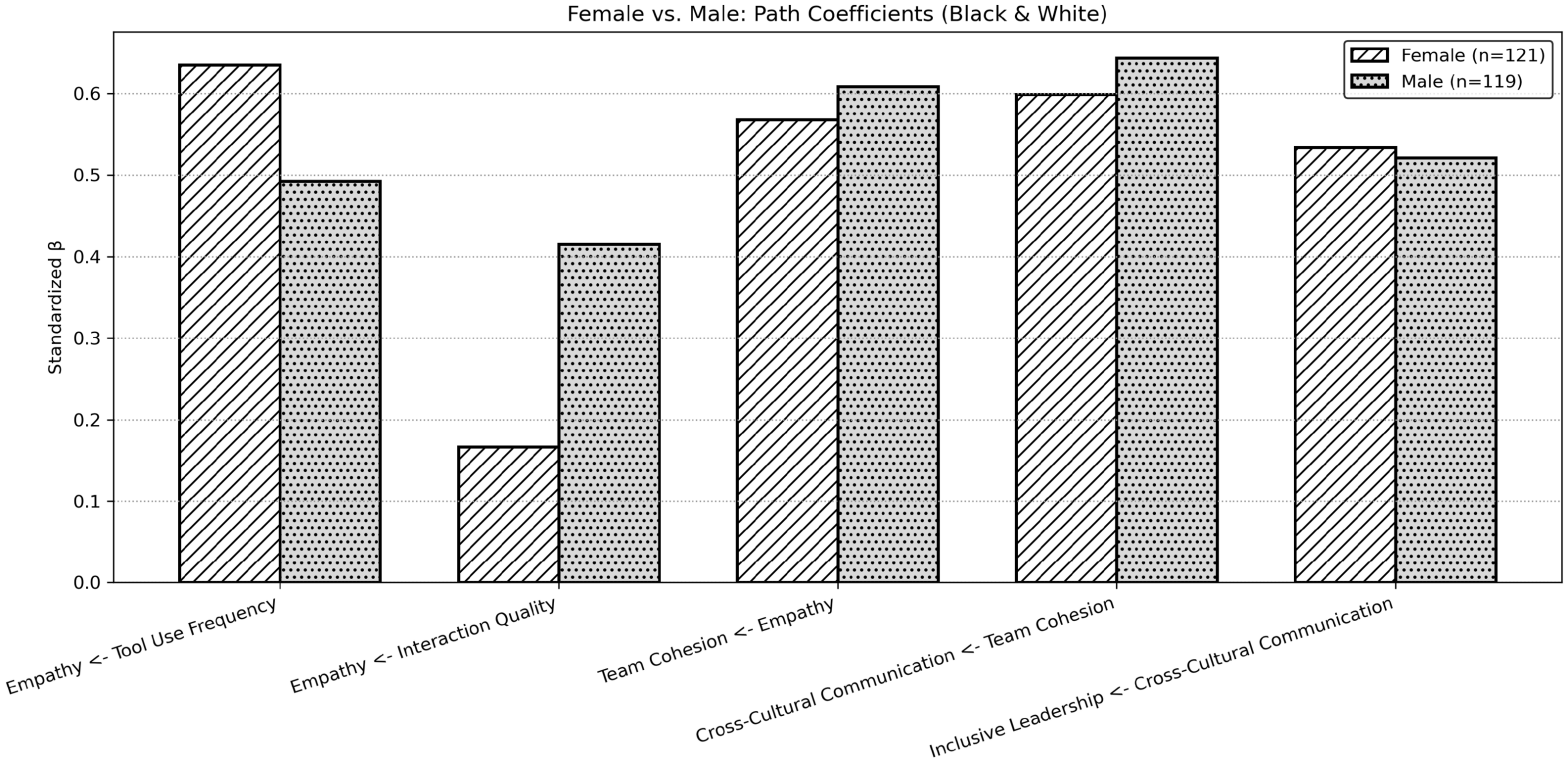
Female vs. male standardized path coefficients.

As reported in Tables 9, 10 and illustrated in Figure 4, the estimated path coefficients from empathy to team cohesion, from team cohesion to cross-cultural communication, and from cross-cultural communication to inclusive leadership were statistically significant for both female and male subgroups. Specifically, empathy was positively associated with team cohesion (female: *β* = 0.568; male: *β* = 0.608, both *p* < 0.001), team cohesion was positively associated with cross-cultural communication (female: *β* = 0.599; male: *β* = 0.644, both *p* < 0.001), and cross-cultural communication was positively associated with inclusive leadership (female: *β* = 0.534; male: *β* = 0.521, both p < 0.001). The explained variance in inclusive leadership was comparable across groups (female: *R*^2^ = 0.285; male: *R*^2^ = 0.272). Differences between subgroups were observed in the paths predicting empathy. Among female respondents, tool usage frequency was positively associated with empathy (*β* = 0.635, *p* < 0.001), whereas interaction quality was not statistically significant (*β* = 0.166, *p* = 0.111). In contrast, among male respondents, both tool usage frequency (*β* = 0.492, *p* < 0.001) and interaction quality (*β* = 0.415, *p* < 0.001) were positively associated with empathy. These results indicate that while the latter segments of the model were statistically supported across both gender groups, the strength and significance of the antecedents of empathy differed between subgroups.

**Table 9:** Multi group path results (female subgroup).

Path	*β* (standardized)	*p* value	Sig.
Empathy ← tool usage frequency	0.622	< 0.001	***
Empathy ← interaction quality	0.164	0.111	
Team cohesion ← empathy	0.608	< 0.001	***
Cross-cultural communication ← team cohesion	0.657	< 0.001	***
Inclusive leadership ← cross-cultural communication	0.492	< 0.001	***
			*R*^2^ (model): 0.285
			Sample size (n): 121

***p < 0.001.

**Table 10:** Multi group path results (male subgroup).

Path	*β* (standardized)	*p* value	Sig.
Empathy ← tool usage frequency	0.492	< 0.001	***
Empathy ← interaction quality	0.415	< 0.001	***
Team cohesion ← empathy	0.608	< 0.001	***
Cross-cultural communication ← team cohesion	0.644	< 0.001	***
Inclusive leadership ← cross-cultural communication	0.521	< 0.001	***
			*R*^2^ (model): 0.272
			Sample size (n): 119

***p < 0.001.

## 5 Discussion

### 5.1 From technology use to inclusive leadership: empirical support for the proposed mechanism

The empirical findings of this study provide clear and consistent support for the core psychological mechanism proposed in the theoretical framework, particularly validating Hypotheses H1 through H3, which together delineate the foundational pathway through which digital collaboration influences inclusive leadership via empathy, team cohesion, and cross-cultural communication. As reported in Tables 6, 8, the frequency of digital collaboration tool use exhibits a strong and robust positive association with empathy, indicating that within multicultural team contexts, sustained engagement with remote collaboration platforms is systematically related to individuals’ capacity for emotional understanding. This result directly supports Hypothesis H1 and aligns with prior work by Romano et al. (2007) on cross-organizational and cross-border collaboration, as well as subsequent research on multicultural virtual teams, which emphasizes that digital media are not limited to the transmission of task-related information but are also capable of conveying social cues and affective signals that shape psychological states and relational perceptions. At the same time, this finding refines efficiency-oriented perspectives that conceptualize collaboration technologies primarily as neutral functional infrastructures for coordination and task execution, perspectives that tend to understate the role of digital tools in shaping emotional processes and social cognition. The present results suggest that even in the absence of face-to-face interaction, frequent and stable digitally mediated engagement can function as an important situational resource for activating empathy, echoing arguments in the digital leadership literature that technology does not necessarily weaken interpersonal emotion but may instead restructure the conditions under which it emerges.

Further support is observed for Hypothesis H2, which posits a positive relationship between empathy and team cohesion. The results demonstrate that empathy exerts a significant positive effect on team cohesion, indicating that the ability to perceive and respond to others’ emotional states contributes directly to the formation of trust, mutual commitment, and relational stability within remote teams. This pattern is consistent with relational leadership theory and social identity approaches, which emphasize that shared emotional understanding facilitates the transformation of individual-level perceptions into collective bonds. It also resonates with prior studies on multicultural teams that identify empathy as a critical antecedent of trust formation, particularly in contexts characterized by cultural heterogeneity and reduced physical proximity. Importantly, the present findings extend this literature by showing that team cohesion is not merely the outcome of formal structures or institutional arrangements, but is deeply rooted in everyday emotional experiences accumulated through ongoing interaction. This relational interpretation is especially salient in multicultural remote teams, where cultural differences and physical distance tend to amplify uncertainty, thereby making empathy a key mechanism for converting individual psychological perceptions into stable team-level relationships. Consistent support is also found for Hypothesis H3, which proposes that team cohesion facilitates effective cross-cultural communication. As shown in Table 8, team cohesion significantly predicts cross-cultural communication effectiveness, suggesting that cohesive relational environments provide a supportive context for clearer, more reciprocal, and more accurate exchanges across cultural boundaries. This finding complements research that has traditionally attributed cross-cultural communication outcomes to language proficiency, media richness, or individual cultural knowledge by demonstrating that communication effectiveness is deeply embedded in relational structures characterized by trust and shared expectations. By empirically linking team cohesion to cross-cultural communication within digitally mediated work environments, this study extends cross-cultural communication theory beyond face-to-face interaction settings and demonstrates that relational mechanisms remain fundamental even under conditions of technological mediation. The consistent support for Hypotheses H1 through H3 therefore establishes the stability of the early stages of the proposed mechanism and shows that digital collaboration tools are embedded within a sequence of psychological and relational processes that form the necessary foundation for subsequent leadership-related outcomes examined later in the analysis.

### 5.2 Digital collaboration as a psychological enabler rather than a technical tool

The results further clarify how inclusive leadership emerges from digitally mediated collaboration by highlighting the conditional and relational nature of the mediation process proposed in Hypotheses H4 through H6. Although cross cultural communication shows a statistically significant relationship with inclusive leadership, its effect size is notably weaker than those observed in earlier stages of the mechanism, suggesting that communication alone may be insufficient to generate strong perceptions of inclusiveness in multicultural remote teams. This finding offers qualified support for Hypothesis H4 and invites a more nuanced reading of prior work that positions cross cultural communication as a central driver of inclusive leadership. For example, research on multicultural leadership has long emphasized communication competence as a defining leadership capability, particularly in culturally diverse teams (Halverson and Tirmizi, 2008; Williams, 2008). However, the present findings indicate that under conditions of digital mediation, communication effectiveness acquires leadership relevance primarily when embedded within a broader relational context. In remote environments where interaction is technologically filtered and cultural ambiguity is heightened, communication appears less capable of independently shaping leadership perceptions and more dependent on preexisting emotional alignment and relational trust. In this sense, the results refine rather than contradict prior communication-centered perspectives by suggesting that cross cultural communication functions as a necessary but not sufficient condition for inclusive leadership.

More compelling evidence is provided for Hypotheses H5 and H6, which posit that empathy influences inclusive leadership through a sequential mediation process involving team cohesion and cross cultural communication. The empirical results indicate that empathy does not exert its influence on inclusive leadership directly, but instead operates as an initiating psychological resource that activates relational processes within the team. This pattern is consistent with relational and process-oriented views of leadership, which conceptualize leadership outcomes as emergent properties of ongoing social interaction rather than as direct expressions of individual traits (Nakamura and Milner, 2023; Calvert, 2018). Empathy strengthens team cohesion by fostering mutual trust and emotional safety, and this cohesive relational climate, in turn, facilitates more effective cross cultural communication. Only when these intermediate relational conditions are established does communication translate into perceptions of inclusive leadership. This sequential structure resonates with Broome’s (1991) relational approach to intercultural communication, which emphasizes shared meaning and mutual recognition over information exchange, and extends it to digitally mediated work contexts. At the same time, the asymmetry observed across the mediation chain suggests important theoretical boundaries. While empathy and team cohesion demonstrate strong and stable associations, the final link between cross cultural communication and inclusive leadership remains comparatively weaker. This pattern implies that communication may act less as a primary causal force and more as a relational amplifier that conditions how emotional and social bonds are interpreted as leadership behaviors. Such an interpretation aligns with recent work on digital and hybrid leadership, which argues that technology reshapes the pathways through which leadership is recognized rather than simply enhancing or diminishing leadership itself (Cortellazzo et al., 2019; Hincapie and Costa, 2024). By empirically demonstrating that inclusive leadership in multicultural remote teams emerges through a cumulative psychological sequence rather than through isolated communicative acts, the present study reinforces Hypotheses H5 and H6 and contributes to a more relationally grounded understanding of inclusive leadership under digital conditions.

### 5.3 When inclusion works differently: gendered pathways and contextual constraints in remote teams

The empirical evidence provides support for Hypothesis H6 by demonstrating that empathy, team cohesion, and cross-cultural communication jointly form a statistically significant sequential mediating pathway between digital collaboration tool use and inclusive leadership. As shown in Tables 6–8, the indirect effect through this ordered chain is robust across analytical approaches, including chain mediation and bootstrap resampling, indicating that the proposed sequence represents a plausible psychological process in multicultural remote teams. This pattern is consistent with relational perspectives in intercultural communication, which emphasize that effective cross-cultural interaction is unlikely to translate into leadership meaning unless it is embedded within affective understanding and stable team relationships, a position articulated early by Broome (1991) and echoed more recently in work on empathic communication and inclusive leadership by Nakamura and Milner (2023). From this perspective, the findings suggest that inclusive leadership perceptions emerge not from isolated communicative acts but from a cumulative relational process in which emotional attunement and cohesion create the conditions under which communication gains leadership relevance.

At the same time, the support for H6 should be interpreted as conditional rather than universal. First, the statistical significance of the sequential pathway does not imply that the mechanism operates with equal strength across all organizational or cultural contexts. The magnitude of downstream effects observed in Tables 6, 8 suggests that the contribution of cross-cultural communication to inclusive leadership may be constrained by situational factors, such as power asymmetries, cultural norms regarding leadership legitimacy, or the degree of task interdependence within teams. Second, the relatively strong correlations among mediators raise the possibility of partial conceptual overlap or shared variance, indicating that empathy, cohesion, and communication may function less as discrete stages than as interrelated dimensions of a broader relational climate. This interpretation introduces a productive tension with more linear process models and cautions against treating the sequential mediation as a deterministic pathway.

## 6 Conclusion

This study examined how digital collaboration tools shape inclusive leadership in multicultural remote teams by identifying the psychological processes through which technology use is translated into leadership perceptions. Drawing on social information processing theory and emotional contagion theory, the findings demonstrate that digital collaboration tools influence inclusive leadership not directly, but through a sequential psychological pathway involving empathy, team cohesion, and cross-cultural communication. Empirically, the results show that the frequency of digital tool use provides a more stable foundation for empathy formation than perceived interaction quality, suggesting that repeated and predictable digitally mediated contact creates the situational conditions under which emotional understanding can emerge in remote and culturally diverse settings. Empathy, in turn, strengthens team cohesion, which facilitates more effective cross-cultural communication, and only through this ordered relational process does inclusive leadership become salient. This evidence advances existing leadership research by shifting attention away from static leadership traits or institutional prescriptions toward micro-level socio-emotional processes embedded in everyday digital interaction. At the same time, multi-group analyses indicate that although the overall structure of this mechanism is stable across genders, the strength of specific links differs, particularly at the entry stage of empathy formation, suggesting that psychological routes to inclusive leadership are contingent rather than universal. Together, these findings contribute theoretically by integrating digital collaboration research with inclusive leadership theory, methodologically by validating a sequential mediation model in a multicultural remote work context, and practically by cautioning organizations against equating technological adoption with relational effectiveness. Future research may build on these results by examining the temporal development of empathy and cohesion under sustained tool use, identifying contextual boundary conditions such as cultural distance or task interdependence, and testing whether deliberate interventions in digital interaction patterns can activate the psychological mechanisms identified in this study.

## 7 Limitations and contribution

Although this study provides a systematic examination of the psychological mechanisms linking digital collaboration tools, empathy, team cohesion, and inclusive leadership, its findings should be interpreted within the specific research context. The sample was primarily drawn from participants operating in remote and hybrid work settings within Chinese and Chinese-speaking environments. While this context allows for the investigation of multicultural interaction, it also implies a relatively bounded cultural setting that may shape the observed relationships. In addition, the cross-sectional design enables the identification of stable and significant structural associations among key variables, but does not capture the dynamic evolution of these psychological and relational mechanisms over time. Future research could therefore benefit from adopting a time-lagged cross-sectional or longitudinal design, which would allow for stronger causal inferences and a more robust examination of how digital collaboration, empathy, and relational processes unfold and reinforce one another over time. Moreover, although gender-based analysis reveals meaningful variation in pathway strength across groups, gender represents only one dimension of individual and structural differentiation, and does not fully account for organizational hierarchies, role configurations, or broader cultural value orientations that may condition leadership processes. Importantly, these boundaries do not diminish the contribution of the study; rather, they help delineate the scope within which its conclusions are most appropriately understood. The central contribution of this research lies in demonstrating that inclusive leadership does not emerge from a single institutional arrangement, leadership style, or technological condition, but is instead a socially and emotionally constructed outcome that unfolds through everyday digital interaction. In contrast to prior work that treats digital tools as neutral media or attributes leadership behavior primarily to individual traits, this study shows that the leadership effects of digital collaboration depend on whether such tools can consistently activate emotional understanding, foster relational bonds, and provide a foundation of trust for cross-cultural interaction. At the same time, the findings caution against viewing this mechanism as automatic or universally applicable, as its effectiveness is likely contingent upon organizational culture, communication norms, and situational structures. By empirically validating this psychological–relational pathway, the study offers a more nuanced and explanatory perspective on the formation of inclusive leadership in remote and hybrid work contexts, while providing a clear theoretical point of departure for future research to extend, refine, and contextualize this mechanism across diverse organizational and cultural settings.

## Data Availability

The raw data supporting the conclusions of this article will be made available by the authors, without undue reservation.
